# Adipocytes Impair Mitoxantrone Cytotoxicity Against Acute Lymphoblastic Leukemia

**DOI:** 10.1002/jha2.70140

**Published:** 2025-08-30

**Authors:** Michael Cohen, Etan Orgel, Jessica Nevarez‐Mejia, Ting Chen, Michael Neely, Stan Louie, Steven D. Mittelman

**Affiliations:** ^1^ Division of Pediatric Endocrinology UCLA Children's Discovery and Innovation Institute David Geffen School of Medicine University of California Los Angeles Los Angeles California USA; ^2^ Cancer and Blood Disease Institute Children's Hospital Los Angeles Los Angeles California USA; ^3^ Department of Pediatrics Keck School of Medicine University of Southern California Los Angeles California USA; ^4^ Laboratory of Applied Pharmacokinetics and Bioinformatics The Saban Research Institute Children's Hospital Los Angeles Los Angeles California USA; ^5^ Mann School of Pharmacy and Pharmaceutical Sciences Keck School of Medicine University of Southern California Los Angeles California USA

**Keywords:** acute leukemia, chemotherapy, drug resistance, pharmacokinetics

## Abstract

**Introduction:**

Obesity contributes to poorer clinical outcomes in patients with acute lymphoblastic leukemia. We have shown that adipocytes cause anthracycline resistance by absorbing and metabolizing them into less‐active alcohol metabolites.

**Methods:**

We hypothesized that mitoxantrone, which has a similar cytotoxic mechanism to anthracyclines but is metabolized through different pathways, might overcome this adipocyte‐mediated chemoresistance. We treated human BV173 acute lymphoblastic leukemia cells with daunorubicin and mitoxantrone that had been incubated with or without 3T3‐L1 adipocytes.

**Results:**

Contrary to our hypothesis, adipocytes induced similar chemoresistance to both drugs.

**Conclusion:**

Mitoxantrone is unlikely to be an attractive alternative to overcome adipocyte‐mediated anthracycline resistance in patients with obesity.

**Trial Registration:**

The authors have confirmed clinical trial registration is not needed for this submission.

## 1 Introduction

Obesity contributes to a higher incidence and mortality of multiple types of cancer [1]. Similar findings have been made in children and adolescents with acute lymphoblastic leukemia (ALL), the most common pediatric cancer [2, 3]. Youth who present with obesity at the time of ALL diagnosis have a significantly increased risk of relapse [2]. Further, a longer duration of obesity during intensive chemotherapy exerts a continued adverse effect on survival [4]. To explore the biology of this adverse influence on ALL chemoresistance and relapse, we have developed several preclinical models, including in vitro with adipocytes and in vivo using obese mice with syngeneic ALL. From these, we were able to recapitulate our clinical findings, where obese mice treated with either vincristine or l‐asparaginase have a poorer outcome than nonobese mice [5, 6]. We have since identified multiple mechanisms by which adipocytes and adipose tissue interact with ALL cells and contribute to chemotherapy resistance [5, 6, 7, 8, 9, 10, 11], explaining how obesity makes leukemia harder to cure.

Anthracycline chemotherapies are a core component of the critical induction phase in ALL regimens. Concerningly, obesity during induction is associated with poorer disease response, with a >2‐fold increased risk for residual leukemia [12]. One mechanism by which adipocytes protect ALL cells during intensive chemotherapy is through the absorption and metabolism of anthracyclines such as daunorubicin [9]. Fat cells express high concentrations of aldo‐ketoreductase (AKR) and carbonyl reductase (CBR) enzymes, which are responsible for metabolizing daunorubicin and doxorubicin to their less cytotoxic alcohol forms, daunorubicinol and doxorubicinol, respectively. These processes inactivate the anthracycline in the adipocyte microenvironment, likely contributing to poor disease response and eventual relapse risk.

**FIGURE 1:**
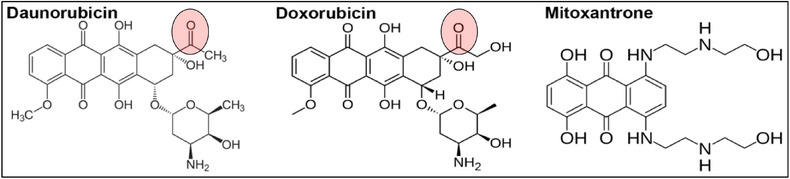
Molecular structure of daunorubicin, doxorubicin, and mitoxantrone. Pink circles indicate carbonyl groups reduced by AKR and CBR to form the alcohol metabolite (adapted from [13]).

Mitoxantrone is an anthracenedione with a similar mechanism of action as anthracyclines. Unlike anthracyclines, mitoxantrone is not metabolized by AKR or CBR because it lacks the carbonyl moiety (Figure 1). Therefore, we hypothesized that mitoxantrone could be an attractive alternative to daunorubicin for patients with obesity, as it would retain better cytotoxicity in an adipocyte‐rich microenvironment.

## 2 Methods

To test this hypothesis, we incubated various concentrations of daunorubicin (Selleck Chemicals) and mitoxantrone (MedChemExpress, Monmouth Junction, NJ) in RPMI media for 24 h in 37°C cell culture incubators either alone or above a monolayer of differentiated 3T3‐L1 adipocytes. Media was collected, and BV173 human ALL cells (ATCC, Manassas, VA) were then cultured in these media for 72 h. Viable cells were quantified on a Countess (Thermo Fisher Scientific, Canoga Park, CA) using trypan blue exclusion (Table S1).

## 3 Results

**FIGURE 2:**
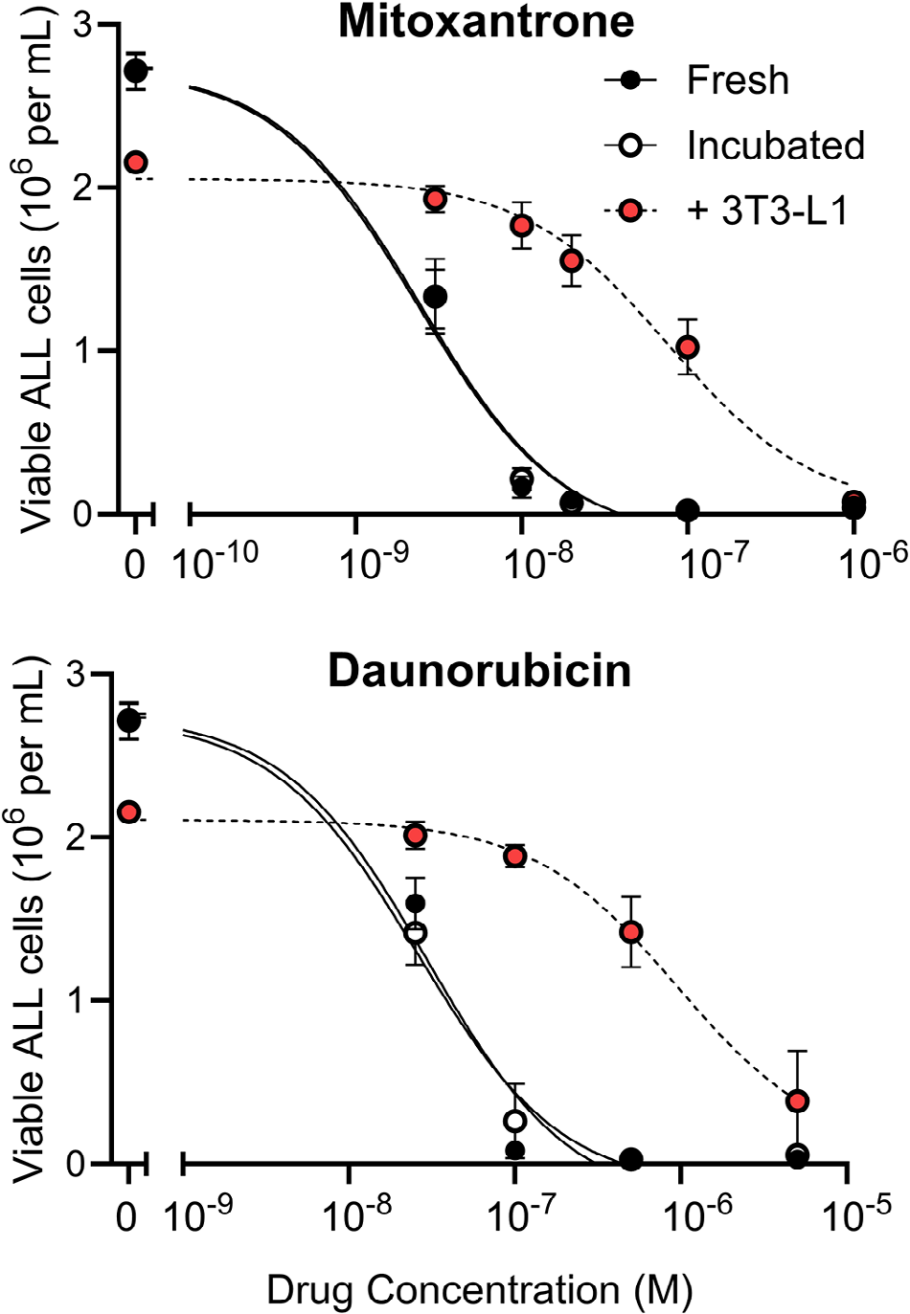
Dose response of BV173 ALL cells to mitoxantrone (top) and daunorubicin (bottom). *N* = 7. Error bars indicate standard error of the mean (SEM).

Both daunorubicin and mitoxantrone killed ALL cells in a dose‐dependent manner, which was not impacted by 24‐h incubation alone at 37°C (Figure 2). The half‐maximal effective concentration (EC_50_) was calculated by fitting aggregate results to a three‐parameter log (inhibitor) versus response curve using least squares (GraphPad Prism 10.2.3). Mitoxantrone showed about ten‐fold higher cytotoxicity compared to daunorubicin. Incubating either drug over 3T3‐L1 adipocytes resulted in a significant loss of cytotoxicity by >30‐fold. The EC_50_ of mitoxantrone was 2.3‐2.4 nM, while incubation over adipocytes increased the EC_50_ to 75.5 nM (31.5‐32.8×). Similarly, the EC_50_ of daunorubicin increased from 26–30 to 950 nM after adipocyte exposure (31.7–36.5×).

## 4 Discussion

Mitoxantrone acts via a similar mechanism to anthracyclines but has more potent anti‐leukemic activity [14]. For relapsed pediatric ALL, mitoxantrone was proven effective for salvage therapy in the UK ALL R3 trial [14] and subsequently incorporated into the Children's Oncology Group AALL1331 re‐induction phase [15]. In fact, metabolism of mitoxantrone via cellular peroxidases or cytochrome P450 enzymes enhances the cytotoxic properties of mitoxantrone [16]. Therefore, we hypothesized that this drug could be a more effective alternative to daunorubicin to overcome adipose tissue‐mediated daunorubicin resistance.

These findings confirmed the increased cytotoxicity of mitoxantrone on ALL cells as compared to daunorubicin, but unfortunately demonstrated equal susceptibility of mitoxantrone to adipocyte chemoprotection. Thus, absorption and sequestration of anthracenediones and anthracyclines from the microenvironment may be the primary mechanism by which adipocytes reduce local cytotoxicity to ALL. For anthracyclines, adipocyte metabolism to a less toxic alcohol metabolite is likely additive but not necessary to reduce drug efficacy. However, because our study did not include measurements of these drugs and their metabolites, we cannot definitively test these conclusions. Further, in vivo studies would be necessary to test whether adipose tissue affects these drugs differently in situ. In any case, our results do not support the use of mitoxantrone as an alternative to anthracyclines to overcome the adverse effects of adipocytes and protect ALL cells from chemotherapy. Other strategies are needed to address the poorer outcomes that youth with obesity suffer in contemporary ALL trials.

## Author Contributions

MC, EO, SL, MN, and SDM conceptualized this project; MC, JN‐M, TC, and SL performed the investigations; SDM acquired funding, supervised the project and wrote the original draft. All authors reviewed and edited the manuscript.

## Ethics Statement

The authors have nothing to report.

## Consent

The authors have nothing to report.

## Conflicts of Interest

EO is a consultant for Jazz Pharmaceuticals.

## Supporting information




**Supporting File 1**. jha270140‐sup‐0001‐SuppMat.pdf

## Data Availability

All data is included in supporting files.
