# Supplementary material for: Adipocytes Impair Mitoxantrone Cytotoxicity Against Acute Lymphoblastic Leukemia
Source: EJHaem. 2025 Aug 30;6(5):e70140. doi: 10.1002/jha2.70140 (PMC12397950; doi:10.1002/jha2.70140)
Supplement: Supplementary file 1 — Supporting File 1. jha270140‐sup‐0001‐SuppMat.pdf [file JHA2-6-e70140-s001.pdf]

Drug	Dose (nM)	log dose	Fresh							Incubated							Incubated over 3T3-L1						
			n1	n2	n3	n4	n5	n6	n7	n1	n2	n3	n4	n5	n6	n7	n1	n2	n3	n4	n5	n6	n7
control	0	--	2.395	2.330	2.475	3.010	2.845	2.950	2.950	2.440	2.455	2.505	2.860	2.690	3.290	2.765	2.020	1.985	2.295	2.017	2.280	2.360	2.140
mitoxantrone	3	0.48	1.325	1.215	0.698	1.730	1.630	ND	ND	1.500	0.579	1.040	1.820	1.725	ND	ND	1.940	1.680	1.870	2.000	2.170	ND	ND
mitoxantrone	10	1	0.317	0.466	0.035	0.195	0.114	0.023	0.018	0.372	0.407	0.085	0.330	0.311	0.009	0.006	1.295	1.700	1.275	1.793	2.055	2.250	2.010
mitoxantrone	20	1.30	0.190	0.225	0.047	0.018	0.088	0.035	0.026	0.232	0.086	0.029	0.038	0.079	0.012	0.009	0.823	1.340	1.290	1.953	1.885	1.840	1.737
mitoxantrone	100	2	ND	ND	0.018	0.018	0.039	0.070	0.041	ND	ND	0.026	0.012	0.018	0.029	0.035	ND	ND	0.479	1.052	1.543	1.037	1.004
mitoxantrone	1000	3	ND	ND	ND	ND	ND	0.021	0.044	ND	ND	ND	ND	ND	0.033	0.044	ND	ND	ND	ND	ND	0.084	0.070
daunorubicin	25	1.4	1.770	0.846	2.050	1.985	1.350	1.695	1.480	1.825	0.305	1.755	1.670	1.470	1.310	1.575	1.965	1.773	1.883	1.993	2.360	2.113	ND
daunorubicin	100	2	0.065	0.029	0.038	0.158	0.088	0.050	0.150	0.079	0.041	0.024	0.006	0.035	0.029	1.640	1.940	1.783	2.030	1.550	2.055	1.830	2.010
daunorubicin	500	2.7	ND	0.003	0.018	0.027	0.067	0.053	ND	ND	0.021	0.015	0.033	0.053	0.026	ND	ND	0.346	1.603	1.595	1.653	1.593	1.737
daunorubicin	5000	3.7	ND	ND	ND	ND	0.021	0.035	ND	ND	ND	ND	ND	0.059	0.056	ND	ND	ND	ND	ND	0.067	0.088	1.004

ND = not done
